# Complete Genome Sequence of Klebsiella pneumoniae Siphophage Seifer

**DOI:** 10.1128/MRA.01289-19

**Published:** 2019-11-14

**Authors:** Adam J. Salazar, Lauren Lessor, Chandler O’Leary, Jason Gill, Mei Liu

**Affiliations:** a Center for Phage Technology, Texas A&M University, College Station, Texas, USA; DOE Joint Genome Institute

## Abstract

Carbapenemase-producing Klebsiella pneumoniae poses a significant public health threat due to its resistance to antibiotics. Siphophage Seifer was isolated and characterized as part of an effort to develop phage therapeutics to control this pathogen. This report describes the complete genome sequence of phage Seifer, which is a distant member of the χ-like siphovirus phage cluster.

## ANNOUNCEMENT

Klebsiella pneumoniae is an opportunistic human pathogen often linked to hospital-acquired infections (1). K. pneumoniae strains that carry the plasmid-borne and highly mobile K. pneumoniae carbapenemases (*bla*_KPC_) are of special concern due to their ability to degrade carbapenem antibiotics (1–3). Phages targeting KPC-positive K. pneumoniae could be used as alternatives to antibiotic treatments.

Phage Seifer was isolated from a wastewater sample collected from Brazos County, TX, in 2015 using a KPC-positive K. pneumoniae clinical isolate of sequence type 258 as the host. Host bacteria were cultured on tryptic soy broth or agar (Difco) at 37°C with aeration. Phages were cultured and propagated using the soft-agar overlay method (4). The phage was identified as a siphophage using negative-stain transmission electron microscopy performed at the Texas A&M University Microscopy and Imaging Center as described previously (5). Phage genomic DNA was prepared using a modified Promega Wizard DNA cleanup kit protocol as described previously (5). Pooled indexed DNA libraries were prepared using the Illumina TruSeq Nano LT kit, and the sequence was obtained from the Illumina MiSeq platform using the MiSeq V2 500-cycle reagent kit following the manufacturer’s instructions, producing 697,877 paired-end 250-bp reads for the index containing the phage Seifer genome. FastQC 0.11.5 (https://www.bioinformatics.babraham.ac.uk/projects/fastqc/) was used to quality control the reads. The reads were trimmed with FastX Toolkit 0.0.14 (http://hannonlab.cshl.edu/fastx_toolkit/download.html) before being assembled using SPAdes 3.5.0 (6). Contig completion was confirmed with PCR using primers (5′-TGTATCCTACGCTCGTTCCC-3′, 5′-CCGATTATGACGCCGCTATG-3′) facing off the ends of the assembled contig and Sanger sequencing of the resulting product, with the contig sequence manually corrected to match the resulting Sanger sequencing read. GLIMMER 3.0 (7) and MetaGeneAnnotator 1.0 (8) were used to predict protein coding genes with manual verification, and tRNA genes were predicted with ARAGORN 2.36 (9). Rho-independent terminators were identified via TransTerm (http://transterm.cbcb.umd.edu/). Sequence similarity searches were performed using BLASTp 2.2.28 (10) with a maximum expectation cutoff of 0.001 against the NCBI nonredundant (nr), UniProt Swiss-Prot (11), and TrEMBL databases. InterProScan 5.15-54.0 (12), LipoP (13), and TMHMM 2.0 (14) were used to predict protein function. All analyses were conducted at default settings via the CPT Galaxy (15) and WebApollo (16) interfaces (https://cpt.tamu.edu/galaxy-pub).

Phage Seifer has a complete genome of 58,197 bp assembled at 212-fold coverage. It has a GC content of 56% and a coding density of 95%. Seifer is a distant member of the χ-like phage cluster (17), with similarity to phage χ in the left arm of the genome. Out of 82 total predicted proteins in phage Seifer, 43 and 45 proteins show similarity to proteins encoded by *Salmonella* phage χ (GenBank accession no. NC_025442) (18, 19) and *Escherichia* phage Utah (GenBank accession no. KY014601) (20), respectively, based on BLASTp with an E value of <0.001. The 12-bp 5′ extended overhang sequences (5′-GGTGCGCAGAGC-3′) in phages χ and Utah were identified in phage Seifer at the beginning of the genome. As determined by BLASTn against the NCBI nucleotide (nt) database, Seifer is closely related to *Klebsiella* phage Soft (GenBank accession no. MN106244) at the nucleotide level by sharing 85% overall identity (E value, 0).

### Data availability.

The genome sequence of phage Seifer was submitted to GenBank under the accession no. MH817999. The associated BioProject, SRA, and BioSample numbers are PRJNA222858, SRR8556780, and SAMN10904482, respectively.

## References

[1] Satlin MJ, Chen L, Patel G, Gomez-Simmonds A, Weston G, Kim AC, Seo SK, Rosenthal ME, Sperber SJ, Jenkins SG, Hamula CL, Uhlemann AC, Levi MH, Fries BC, Tang YW, Juretschko S, Rojtman AD, Hong T, Mathema B, Jacobs MR, Walsh TJ, Bonomo RA, Kreiswirth BN 2017 Multicenter clinical and molecular epidemiological analysis of bacteremia due to carbapenem-resistant Enterobacteriaceae (CRE) in the CRE epicenter of the United States. Antimicrob Agents Chemother 61:e02349-16. doi:10.1128/AAC.02349-16. 28167547 PMC5365653

[2] Sanchez GV, Master RN, Clark RB, Fyyaz M, Duvvuri P, Ekta G, Bordon J 2013 Klebsiella pneumoniae antimicrobial drug resistance, United States, 1998–2010. Emerg Infect Dis 19:133–136. doi:10.3201/eid1901.120310. 23260464 PMC3557979

[3] Tumbarello M, Viale P, Viscoli C, Trecarichi EM, Tumietto F, Marchese A, Spanu T, Ambretti S, Ginocchio F, Cristini F, Losito AR, Tedeschi S, Cauda R, Bassetti M 2012 Predictors of mortality in bloodstream infections caused by Klebsiella pneumoniae carbapenemase-producing K. pneumoniae: importance of combination therapy. Clin Infect Dis 55:943–950. doi:10.1093/cid/cis588. 22752516

[4] Adams MK 1959 Bacteriophages. Interscience Publishers, Inc, New York, NY.

[5] Gill JJ, Berry JD, Russell WK, Lessor L, Escobar-Garcia DA, Hernandez D, Kane A, Keene J, Maddox M, Martin R, Mohan S, Thorn AM, Russell DH, Young R 2012 The Caulobacter crescentus phage phiCbK: genomics of a canonical phage. BMC Genomics 13:542. doi:10.1186/1471-2164-13-542. 23050599 PMC3556154

[6] Bankevich A, Nurk S, Antipov D, Gurevich AA, Dvorkin M, Kulikov AS, Lesin VM, Nikolenko SI, Pham S, Prjibelski AD, Pyshkin AV, Sirotkin AV, Vyahhi N, Tesler G, Alekseyev MA, Pevzner PA 2012 SPAdes: a new genome assembly algorithm and its applications to single-cell sequencing. J Comput Biol 19:455–477. doi:10.1089/cmb.2012.0021. 22506599 PMC3342519

[7] Delcher AL, Harmon D, Kasif S, White O, Salzberg SL 1999 Improved microbial gene identification with GLIMMER. Nucleic Acids Res 27:4636–4641. doi:10.1093/nar/27.23.4636. 10556321 PMC148753

[8] Noguchi H, Taniguchi T, Itoh T 2008 MetaGeneAnnotator: detecting species-specific patterns of ribosomal binding site for precise gene prediction in anonymous prokaryotic and phage genomes. DNA Res 15:387–396. doi:10.1093/dnares/dsn027. 18940874 PMC2608843

[9] Laslett D, Canback B 2004 ARAGORN, a program to detect tRNA genes and tmRNA genes in nucleotide sequences. Nucleic Acids Res 32:11–16. doi:10.1093/nar/gkh152. 14704338 PMC373265

[10] Camacho C, Coulouris G, Avagyan V, Ma N, Papadopoulos J, Bealer K, Madden TL 2009 BLAST+: architecture and applications. BMC Bioinformatics 10:421. doi:10.1186/1471-2105-10-421. 20003500 PMC2803857

[11] The UniProt Consortium. 2018 UniProt: the universal protein knowledgebase. Nucleic Acids Res 46:2699. doi:10.1093/nar/gky092. 29425356 PMC5861450

[12] Jones P, Binns D, Chang HY, Fraser M, Li W, McAnulla C, McWilliam H, Maslen J, Mitchell A, Nuka G, Pesseat S, Quinn AF, Sangrador-Vegas A, Scheremetjew M, Yong SY, Lopez R, Hunter S 2014 InterProScan 5: genome-scale protein function classification. Bioinformatics 30:1236–1240. doi:10.1093/bioinformatics/btu031. 24451626 PMC3998142

[13] Juncker AS, Willenbrock H, Von Heijne G, Brunak S, Nielsen H, Krogh A 2003 Prediction of lipoprotein signal peptides in Gram-negative bacteria. Protein Sci 12:1652–1662. doi:10.1110/ps.0303703. 12876315 PMC2323952

[14] Krogh A, Larsson B, von Heijne G, Sonnhammer EL 2001 Predicting transmembrane protein topology with a hidden Markov model: application to complete genomes. J Mol Biol 305:567–580. doi:10.1006/jmbi.2000.4315. 11152613

[15] Cock PJ, Gruning BA, Paszkiewicz K, Pritchard L 2013 Galaxy tools and workflows for sequence analysis with applications in molecular plant pathology. PeerJ 1:e167. doi:10.7717/peerj.167. 24109552 PMC3792188

[16] Lee E, Helt GA, Reese JT, Munoz-Torres MC, Childers CP, Buels RM, Stein L, Holmes IH, Elsik CG, Lewis SE 2013 Web Apollo: a Web-based genomic annotation editing platform. Genome Biol 14:R93. doi:10.1186/gb-2013-14-8-r93. 24000942 PMC4053811

[17] Grose JH, Casjens SR 2014 Understanding the enormous diversity of bacteriophages: the tailed phages that infect the bacterial family Enterobacteriaceae. Virology 468–470:421–443. doi:10.1016/j.virol.2014.08.024. PMC4301999 25240328

[18] Lee JH, Shin H, Choi Y, Ryu S 2013 Complete genome sequence analysis of bacterial-flagellum-targeting bacteriophage chi. Arch Virol 158:2179–2183. doi:10.1007/s00705-013-1700-0. 23605589

[19] Hendrix RW, Ko CC, Jacobs-Sera D, Hatfull GF, Erhardt M, Hughes KT, Casjens SR 2015 Genome sequence of Salmonella phage chi. Genome Announc 3:e01229-14. doi:10.1128/genomeA.01229-14. PMC4342425 25720684

[20] Leavitt JC, Heitkamp AJ, Bhattacharjee AS, Gilcrease EB, Casjens SR 2017 Genome sequence of Escherichia coli tailed phage Utah. Genome Announc 5:e01494-16. doi:10.1128/genomeA.01494-16. 28360173 PMC5374247

